# The composition of cigarette smoke determines inflammatory cell recruitment to the lung in COPD mouse models

**DOI:** 10.1042/CS20130117

**Published:** 2013-10-09

**Authors:** Gerrit John, Katrin Kohse, Jürgen Orasche, Ahmed Reda, Jürgen Schnelle-Kreis, Ralf Zimmermann, Otmar Schmid, Oliver Eickelberg, Ali Önder Yildirim

**Affiliations:** *Comprehensive Pneumology Center, Institute of Lung Biology and Disease, Helmholtz Zentrum München, Member of the German Center for Lung Research, Ingolstädter Landstr. 1, 85764 Neuherberg, Germany; †Joint Mass Spectrometry Centre of Helmholtz Zentrum München and University of Rostock, Comprehensive Molecular Analytics, Helmholtz Zentrum München, Ingolstädter Landstr. 1, 85764 Neuherberg, Germany; ‡Klinikum der Universität München, Max-Lebsche-Platz 31, 81377 München, Germany; §HICE–Helmholtz Virtual Institute of Complex Molecular Systems in Environmental Health-Aerosols and Health

**Keywords:** chronic obstructive pulmonary disease (COPD), inflammation, mainstream, neutrophil, sidestream, smoke, BAL, bronchoalveolar lavage, CC, carbonyl compounds, CMD, count median diameter, CO-Hb, carboxyhaemoglobin, COPD, chronic obstructive pulmonary disease, CS, cigarette smoke, DNPH, 2,4-dinitrophenylhydrazine, GC-SIM-MS, gas-chromatography with selective ion monitoring MS, GM-CSF, granulocyte macrophage colony-stimulating factor, HO, haem oxygenase, H&E, haematoxylin and eosin, HPRT-1, hypoxanthine–guanine phosphoribosyltransferase, IFNβ, interferon β, IL-1β, interleukin 1β, KC, keratinocyte chemoattractant, LPS, lipopolysaccharide, MCP-1, monocyte chemoattractant protein-1, MIP, macrophage inflammatory protein, MMD, mass median diameter, MMP12, matrix metalloproteinase 12, NE, neutrophil elastase, NF-κB, nuclear factor κB, PAH, polycyclic aromatic hydrocarbons, PM, particulate matter, TNF-α, tumour necrosis factor α, TPM, total particulate matter

## Abstract

COPD (chronic obstructive pulmonary disease) is caused by exposure to toxic gases and particles, most often CS (cigarette smoke), leading to emphysema, chronic bronchitis, mucus production and a subsequent decline in lung function. The disease pathogenesis is related to an abnormal CS-induced inflammatory response of the lungs. Similar to active (mainstream) smoking, second hand (sidestream) smoke exposure severely affects respiratory health. These processes can be studied *in vivo* in models of CS exposure of mice. We compared the acute inflammatory response of female C57BL/6 mice exposed to two concentrations [250 and 500 mg/m^3^ TPM (total particulate matter)] of sidestream and mainstream CS for 3 days and interpreted the biological effects based on physico-chemical differences in the gas and particulate phase composition of CS. BAL (bronchoalveolar lavage fluid) was obtained to perform differential cell counts and to measure cytokine release. Lung tissue was used to determine mRNA and protein expression of proinflammatory genes and to assess tissue inflammation. A strong acute inflammatory response characterized by neutrophilic influx, increased cytokine secretion [KC (keratinocyte chemoattractant), TNF-α (tumour necrosis factor α), MIP-2 (macrophage inflammatory protein 2), MIP-1α and MCP-1 (monocyte chemoattractant protein-1)], pro-inflammatory gene expression [KC, MIP-2 and MMP12 (matrix metalloproteinase 12)] and up-regulated GM-CSF (granulocyte macrophage colony-stimulating factor) production was observed in the mainstream model. After sidestream exposure there was a dampened inflammatory reaction consisting only of macrophages and diminished GM-CSF levels, most likely caused by elevated CO concentrations. These results demonstrate that the composition of CS determines the dynamics of inflammatory cell recruitment in COPD mouse models. Different initial inflammatory processes might contribute to COPD pathogenesis in significantly varying ways, thereby determining the outcome of the studies.

## INTRODUCTION

COPD (chronic obstructive pulmonary disease) is a major public health problem and its morbidity and mortality are still rising [1]. Patients suffering from COPD exhibit a constant and accelerated decline in lung function leading to airflow limitation. The pathogenesis of COPD is characterized by severe pathophysiological changes including chronic bronchitis, small airway remodelling, mucus production and the development of emphysaema [2]. However, the exact processes involved in disease pathogenesis are so far not fully understood and treatment can only aim at alleviating symptoms.

CS (cigarette smoke) is a major etiologic factor in the pathogenesis of COPD and other diseases [3]. The health impact of environmental CS is under critical evaluation, due to the risk for non-smokers. Environmental CS is mainly composed of exhaled mainstream CS and sidestream CS, whereby the impact of sidestream CS with respect to environmental CS surpasses that of exhaled mainstream CS [4]. Chronic CS exposure results in severe lung inflammation by initiating a complex inflammatory cascade accompanied by the activation and influx of various inflammatory cells and by the secretion of disease-specific mediators such as cytokines and growth factors [5]. The smoke-induced increase in inflammatory cell influx and responses involves both innate immune cells, with macrophages and neutrophils predominating, and adaptive immune cells, specifically T and B lymphocytes. The numbers of neutrophils, macrophages and lymphocytes are increased in both airways and parenchyma of patients with COPD [6–9]. The progression and severity of COPD are also associated with increasing infiltration of the airways by innate and adaptive immune cells, leading to obstruction of small airways and thickening of the airway wall [10]. Nevertheless, it still remains unclear that inflammatory events and cell types involved in this complex cascade are central for the development of COPD.

In order to elucidate these processes *in vivo*, CS exposure of rodents/mice is a commonly used model. Chronic exposure, that is, up to 4 months or longer, recapitulates most features of COPD by inducing pathophysiological changes in mice similar to those observed in COPD patients, such as small airway remodelling and septal tissue damage/emphysaema. On the other hand, acute CS exposure is mostly useful and employed to study early and more direct effects of smoking on inflammatory responses and cell recruitment in the lung, specifically innate immune cells [11].

Several animal studies have analysed the contribution of neutrophils and macrophages to CS-induced lung inflammation and subsequent emphysema development. It is now generally accepted that in CS models the inflammatory response driving the pathophysiological changes is characterized by two phases: the acute reaction during the first week of CS exposure, which shows a strong neutrophilic influx, and the progressive inflammation after 1 month of exposure consisting of neutrophils, macrophages and lymphocytes [12–14]. Interestingly, it was shown that in CS-exposed mice innate immune cells were sufficient for driving inflammatory processes in the lung thereby initiating COPD development [15,16]. Innate immune cell-specific factors MMP (matrix metalloproteinase) 12 and NE (neutrophil elastase) were even necessary for inducing experimental emphysema after prolonged exposure [17,18]. Although CS-exposed MMP12-knockout mice failed to recruit macrophages and did not develop lung destruction [17], NE was required for neutrophil and monocyte recruitment as well as for the activation of MMP12 after CS exposure [18]. These studies highlight the importance of prompt CS-induced inflammatory mechanisms and innate immune cell activation that potentially contribute to COPD pathogenesis at an early stage of smoke exposure.

However, different smoke exposure models with regards to machines and setups using either mainstream smoke only or a mixture of side- and main-stream smoke (usually consisting of about 90% sidestream smoke and 10% mainstream smoke) have been established and might generate varying results, especially when comparing the degree of CS-induced changes [19]. The dynamics of early lung immune cell reactions to CS was characterized using the mainstream model in acute settings of smoke exposure for a few days [20–22]. But even though sidestream CS is more toxic than mainstream CS [23], data on acute lung inflammation from this model are missing. The only acute sidestream study investigated oxidative DNA damage in mouse heart, liver and lung tissue after a single CS exposure [24].

Interestingly, time- and dose-dependent inflammatory responses after acute mainstream CS exposure have already been described [20–22]. However until now, no study has ever performed a comprehensive comparison of the dynamics of acute lung inflammation between the main- and side-stream CS model with regards to physico-chemical characteristics of CS, specifically concentration, size distribution and chemical composition of TPM (total particulate matter) as well as composition of the gas phase.

Therefore using two different experimental models of smoke exposure (mainstream and sidestream CS) with two TPM concentrations, the present study aimed at investigating the effect of the physico-chemical characteristics of CS on the acute inflammatory response in the lungs of mice after 3 days of CS exposure. We hypothesized that early signs of inflammation would be apparent, but significantly different between the two types of CS. The results were interpreted based on detailed information on concentration, size distribution and chemical composition of TPM as well as on CO concentration.

Interestingly, a strong acute inflammatory response characterized by neutrophilic influx, accompanied by increased cytokine secretion and pro-inflammatory gene expression, and up-regulated GM-CSF (granulocyte macrophage colony-stimulating factor) production was only observed in the mainstream CS model, whereas there was a dampened inflammatory reaction after sidestream exposure, most probably caused by elevated CO concentrations.

## MATERIALS AND METHODS

### Animals and maintenance

Pathogen-free female C57BL/6 mice (8–10-weeks old) were obtained from Charles River and housed in rooms maintained at constant temperature and humidity with a 12 h light cycle. Animals were allowed food and water *ad libitum*. All animal experiments were conducted under strict governmental and international guidelines and were approved by the local government for the administrative region of Upper Bavaria.

### CS exposure and CO monitoring

For both exposure models, smoke was generated from 3R4F Research Cigarettes (Tobacco Research Institute, University of Kentucky, Lexington, KY, U.S.A.). Control mice for all experiments were kept in a filtered air environment.

For sidestream CS exposure, mice were placed in an exposure chamber connected to a TE-10 smoking machine (Teague Enterprises). The machine is adjusted to produce 89% sidestream and 11% mainstream smoke. The chamber atmosphere was monitored to maintain TPM at 250 and 500 mg/m^3^ and mice were exposed to CS for 50 min twice per day for 3 consecutive days.

In the mainstream CS model, mice were exposed to active smoke (100% mainstream smoke) in a manner mimicking natural human smoking habits. The smoke was drawn into the exposure chamber via a membrane pump. Mice were exposed to CS of 250 and 500 mg/m^3^ TPM for 50 min twice per day for 3 consecutive days as described by Eltom et al. [25].

TPM concentrations were monitored by drawing sample air from the exposure chamber through a quartz fibre filter and measuring the total air volume. The TPM mass concentration was obtained via gravimetric analysis of the filters prior to and after exposure (estimated accuracy of approximately 5%). CO concentrations in the exposure chamber were constantly monitored by using a GCO 100 CO Meter (Greisinger Electronic). Levels of arterial blood CO-Hb (carboxyhaemoglobin) in mice 30 min after CS exposure were determined by retro-orbital blood sampling and analysed in an ABL80 FLEX blood gas analyser (Radiometer). All CS exposure experiments were performed with *n*=6 animals per group and were repeated twice.

### CS particle size measurements

The size distribution of the PM (particulate matter) of CS was measured during exposure experiments according to the same protocol as described above, but without mice in the exposure chamber. Sample air was drawn from the sampling port of the exposure chamber (located in the centre near the top of the chamber) and delivered at 0.75 l/min through 3 m of tubing to a scanning mobility particle sizer (SMPS, TSI, Model 3080, TSI) in combination with a CPC (condensation particle counter; Model 3025, TSI). The excess air was resupplied into the exposure system downstream of the exposure chamber. The SMPS is a standard aerosol sizer with a sizing accuracy of up to 2% (here approximately 5%), which measures the size of aerosol particles based on their velocity (mobility) in an electric field [26]. To avoid potential measurement biases because of high particle concentration and high CO/CO_2_ concentrations, the sample air was diluted by a factor of 650 and 400 for the mainstream and sidestream conditions respectively [27]. The SMPS was operated at a sample and sheath flow rate of 0.3 and 1.5 l/min respectively, and the inertial impactor at the inlet of the SMPS was removed to obtain an upper sizing limit of 1000 nm (lower sizing limit, 23 nm). An entire size distribution was obtained within 3 min. For quality assurance, the particle sizing accuracy of the SMPS was verified with size-certified reference particles (230 and 506 nm) and filtre samples were taken for gravimetric analysis of aerosol mass concentrations. For each of the four investigated cases (sidestream and mainstream at 250 and 500 mg/m^3^) 3–5 size distributions were recorded and averaged, since no significant trends were observed after a short equilibration phase of a few minutes. The measured number size distributions between 23 and 1000 nm were converted into length, surface area and mass-weighted size distributions (density of 1 g/cm^3^) and the data were fitted simultaneously on all four moments (number, length, surface area and mass) assuming a lognormal shape with constant geometric standard deviation. Since the median diameters of these moments are directly related by the Hatch–Choate moment conversion equations, this procedure enhances measurement accuracy especially near the upper size range, where a part of the size distribution was outside of the detection limit of the SMPS [28].

### Analysis of organic PM compounds in mainstream and sidestream CS

For organic analyses of the PM of CS, approximately 25 litres of sample air were drawn from the exposure chamber and PM was collected on quartz fibre filters (T293, 25 mm diameter; Munktell). Prior to sampling, the filters were tempered at 500°C for at least 12 h.

The samples were analysed by using a newly developed *in-situ* derivatization and thermal desorption method, which is coupled to GC-MS (gas-chromatography with mass selective detection) [29]. An internal standard mixture (isotope labelled compounds) was spiked on a filter punch (3 mm diameter) prior to analysis for quantification.

### Gas phase analysis of CC (carbonyl compounds) in mainstream and sidestream CS

Carbonyl emissions in the gas phase of CS were sampled using high sample volume DNPH (2,4-dinitrophenylhydrazine) cartridges (Sigma–Aldrich). Parallel samples were collected for each CS type with different flow rates starting form 0.16 to 1.2 l/min using critical nozzles connected to a vacuum pump.

CCs were assessed by GC-SIM-MS (gas-chromatography with selective ion monitoring MS) using DNPH derivatization. Prior to analysis, the cartridges were eluted with 1 ml of acetonitrile and samples were injected into the GC-SIM-MS system for quantitative measurements.

### Animal preparation

At 24 h after the last CS exposure, mice were killed with an overdose of ketamine/xylazine followed by exsanguination. Mice were dissected and BAL (bronchoalveolar lavage) was obtained to perform total and differential cell counts for inflammatory cell recruitment of neutrophils, macrophages and lymphocytes. BAL fluid was used to evaluate cytokine secretion via multiplex analysis. Lung tissue was either shock-frozen in liquid nitrogen to determine tissue mRNA expression or fixed by intratracheal instillation of PBS-buffered 6% (v/v) PFA (paraformaldehyde) and embedded into paraffin for H&E (haematoxylin and eosin) staining.

### Preparation of BAL

The lungs were lavaged by using a cannula inserted into the trachea and instilling the lungs with 4×0.5 ml aliquots of sterile PBS (Gibco). For cytospins, cells were spun down at 400 ***g*** and resuspended in RPMI 1640 medium containing 10% (v/v) FBS (both from Gibco). Total cell counts were determined in a hemocytometer via Trypan Blue exclusion. Maximally 1–2% Trypan Blue-positive cells were detected in both filtered air and CS-exposed animals from the two CS models. Differential cell counts were performed using morphological criteria on May–Grünwald–Giemsa-stained cytospins (200 cells/sample).

### Quantitative real-time RT (reverse transcription)-PCR

Total RNA from lung tissue homogenate was isolated using a peqGOLD Total RNA Kit (Peqlab) according to the manufacturer's instructions. cDNA was synthesized using Random Hexamers and MuLV Reverse Transcriptase (Applied Biosystems). mRNA expression of target genes KC (keratinocyte chemoattractant; CXCL1), TNF-α (tumour necrosis factor α), MIP-2 (macrophage inflammatory protein 2) (CXCL2), MMP12, CD68 and GM-CSF in comparison with housekeeping control HPRT-1 (hypoxanthine–guanine phosphoribosyltransferase 1) was determined using Platinum SYBR Green qPCR SuperMix (Applied Biosystems) on a StepOnePlus™ 96 well Real-Time PCR System (Applied Biosystems). The primers used are listed in Table 1. Relative transcript expression of a gene is given as 2^−Δ*C*_t_^ (Δ*C*_t_=*C*_t_target__−*C*_t_reference__), relative changes compared with control are 2^−ΔΔ*C*_t_^ values (ΔΔ*C*_t_=Δ*C*_t_treated__−Δ*C*_t_control__). Primers were generated using Primer-BLAST software [30].

**Table 1:** Primers used for quantitative real-time PCR of lung tissue

Gene	Forward primer (5′→3′)	Reverse primer (5′→3′)
HPRT-1	CCTAAGATGAGCGCAAGTTGAA	CCACAGGACTAGAACACCTGCTAA
KC (CXCL1)	CCGAGTCATAGCCACAC	GTGCCATCAGAGCAGTCT
TNF-α	CACCACGCTCTTCTGTCT	GGCTACAGGCTTGTCACTC
MIP-2 (CXCL2)	CTGTTGTGGCCAGTGAAC	GCCCTTGAGAGTGGCTAT
MMP12	TGTACCCCACCTACAGATACCTTA	CCATAGAGGGACTGAATGTTACGT
CD68	CCTCCACCCTCGCCTAGT C	TTGGGTATAGGATTCGGATTTGA
GM-CSF	GCCATCAAAGAAGCCCTG	GCGGGTCTGCACACATGTTA

### Multiplex cytokine analysis

Concentrations of secreted cytokines and chemokines IL-1β (interleukin 1β), IL-2, IL-4, IL-5, IL-6, IL-7, IL-9, IL-10, IL-13, IL-17A, GM-CSF, KC (CXCL1), TNF-α, IFNβ (interferon β), MCP-1 (monocyte chemoattractant protein-1; CCL2), MIG (monokine induced by interferon-γ; CXCL9), MIP2 (CXCL2) and MIP-1α (CCL3) in BAL were determined using a magnetic bead-based MILLIPLEX *MAG* multiplex assay (Millipore) and analysed on a Luminex^100^ (Bio-Rad Laboratories). For this assay, BAL fluid was concentrated (10×) by ultrafiltration in Amicon Ultra-0.5 centrifugal filter devices (Millipore).

### Statistics

Results are given as mean values±S.D. One-way ANOVA following Bonferroni post-hoc test was used for all studies with more than two groups, if equal variances and normal distribution was given. Student's unpaired *t* test was performed to compare the concentration of particulate organic compounds [PAHs (polycyclic aromatic hydrocarbons) and alkanes] between two different TPMs. Analyses were conducted using GraphPad Prism 6 software (GraphPad Software).

## RESULTS

### Sidestream CS particles are smaller in size than mainstream CS particles

**Figure 1:**
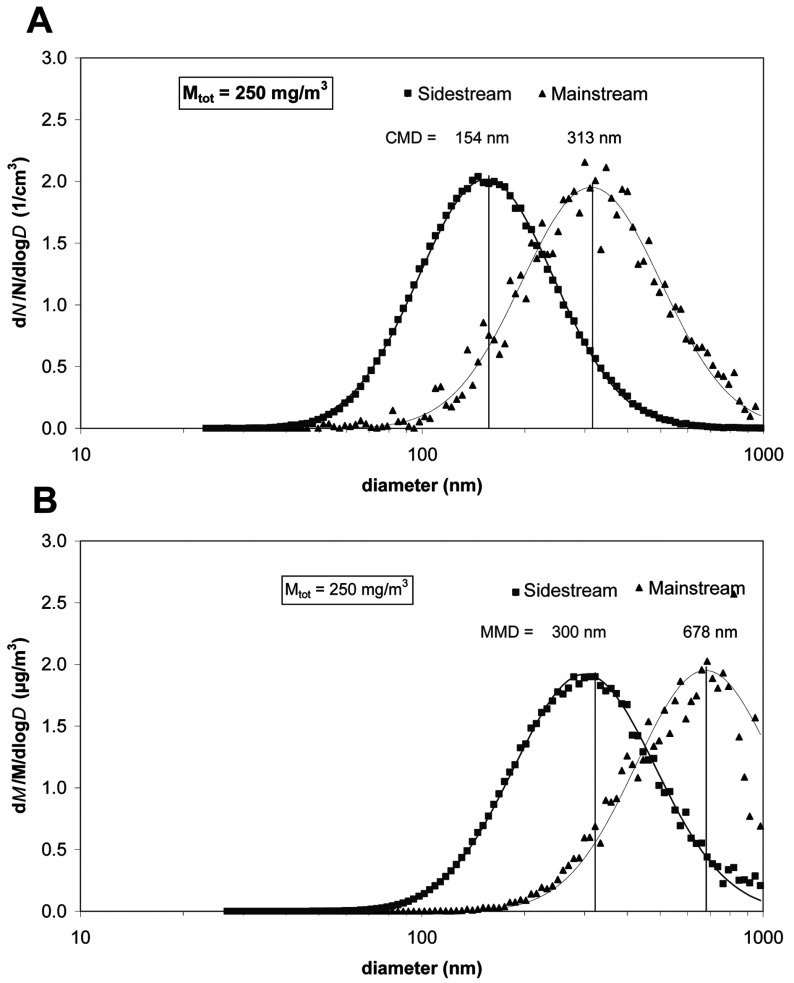
Measured number (A) and mass-based size distribution (B) of CS particles in the exposure chamber at a TPM of 250 mg/m^3^ for both sidestream (square) and mainstream CS (triangle) The symbols represent the measured values and the solid lines represent the corresponding lognormal curve fit of the data. CMD and MMD of the size distributions are also depicted. The size distributions were normalized to their respective total number and total mass concentration.

The size distribution of the PM of CS was determined using an SMPS. Figure 1 depicts the number- and mass-weighted particle size distribution for sidestream and mainstream conditions for a mass concentration of 250 mg/m^3^. A summary of all size distribution data is presented in Table 2.

**Table 2:** Overview of characteristic parameters of the cigarette smoke size distributions for sidestream and mainstream conditions *M*_tot_, mass concentration of total particulate matter in the smoke chamber; σ_g_, geometric S.D. *The reported alveolar lung deposited fraction refers to the specific MMD and is based on *in silico* lung deposition data for mice published by Nadithe et al. [31]. **A breathing frequency of 150/min and a tidal volume of 200 μl were assumed, resulting in an inhaled air volume of 1.8 l/h.

		Sidestream		Mainstream	
Parameter	*M*_tot_ (mg/m^3^)…	250	500	250	500
CMD (nm)		154	189	313	338
σ_g_		1.60	1.56	1.60	1.62
MMD (nm)		300	373	678	756
Lung-deposited particle fraction*		0.11	0.11	0.05	0.05
Lung-deposited particle dose per h (μg/h)**		49.5	99	22.5	45

Sidestream CS particles exhibited a CMD (count median diameter) of 154 nm and were therefore significantly smaller than mainstream CS particles (CMD=313 nm) (Figure 1A). The geometric standard deviation (width of the size distribution) was about 1.60 for all cases (Table 2). Multiplication of each size bin of the number size distribution with the respective (spherical) particle volume (πd^3^/6, *d*=particle diameter) and density (1 g/cm^3^) provided the corresponding mass-weighted size distribution. As depicted in Figure 1(B), the mass-weighted size distribution showed an MMD (mass median diameter) of 300 and 678 nm for sidestream and mainstream CS particles respectively, confirming that mainstream CS particles were approximately two times larger than sidestream CS particles. The increased size of mainstream particles was observed for both concentration settings (250 and 500 mg/m^3^), with 10–20% larger diameters for the 500 mg/m^3^ case (Table 2). It is important to note that even though a significant fraction of the mass-based mainstream size distribution was beyond the upper sizing limit of the SMPS (1000 nm), the reported MMD were still reliable, since the curve fit was performed simultaneously on all moments (number, length, surface area and mass) as described in the Materials and methods section.

From the size distribution parameters provided in Table 2, the biologically relevant lung deposited dose was calculated by multiplying the aerosol mass concentration by the inhaled air volume (1.8 l/h) and the lung-deposited particle fraction (at the MMD) provided by Nadithe et al. [31]. The lung deposited particle dose ranged from 22.5 to 99 μg/h, depending on the aerosol mass concentration and sidestream or mainstream smoke conditions. Although there are several *in silico* models of particle deposition in the lungs of mice, their general trend predicted elevated deposition of 300 nm relative to 700 nm particles, due to enhanced diffusional deposition of 300 nm particles [31].

In summary, mainstream CS particles were approximately two times larger than sidestream CS particles for a given TPM level. Enhancing the TPM level from 250 to 500 mg/m^3^ increased the particle size only moderately (10–20%). This indicates that the type of CS generation (mainstream/sidestream) was more relevant for the observed particle size than the TPM level. Interestingly, the biologically relevant, i.e. lung deposited particle dose for 250 mg/m^3^ TPM sidestream conditions corresponded closely to the pulmonary dose delivered for 500 mg/m^3^ TPM mainstream conditions (Table 2).

### Organic composition of CS particles

A total of 14 relevant PAHs in the PM CS were analysed (Table 3). Sidestream CS showed extremely high concentrations of PAH. The most toxic Benz[a]pyrene [LD_50 (rat)_=50 mg/kg] within the compound class of PAHs were found in potentially harmful concentrations in sidestream CS (1.4 μg/m^3^ at 250 mg/m^3^ TPM and 2.7 μg/m^3^ at 500 mg/m^3^ TPM, with corresponding lung delivered doses of 0.069 and 0.13 μg/kg respectively), whereas the levels below the LOQ (limit of quantification; 1 ng/m^3^) were measured in mainstream CS. The composition of mainstream CS showed no significant differences in concentrations of PAHs with regards to different TPM levels.

**Table 3:** Sidestream and mainstream yields of PAH from two different cigarette smoke concentrations *P* values were calculated using Student's unpaired *t* test. Values in bold are significant. <DL, below detection limit; na, not analysed.

	Sidestream			Mainstream		
Substance (μg/m^3^)	250 mg/m^3^	500 mg/m^3^	*P* value	250 mg/m^3^	500 mg/m^3^	*P* value
Phenanthrene	28.6	55.0	**0.01**	8.6	8.4	0.7
Anthracene	6.1	14.3	**0.01**	1.7	1.5	0.5
Pyrene	11.1	18.2	**0.02**	3.1	2.6	0.1
Fluoranthene	4.7	7.1	**0.01**	2.5	2.4	0.6
Benzo[a]anthracene	2.2	4.0	**0.00**	0.5	<DL	na
Chrysene	7.7	15.0	**0.01**	0.9	<DL	na
Sum Benzofluoranthenes	2.0	4.1	**0.00**	<DL	<DL	na
Benzo[e]pyrene	0.7	1.3	0.35	<DL	<DL	na
Benzo[a]pyrene	1.4	2.7	**0.01**	<DL	<DL	na
Perylene	1.2	2.8	0.11	<DL	<DL	na
Dibenzo[ah]anthracene	<DL	<DL	na	<DL	<DL	na
Indeno[1,2,3-cd]pyrene	1.3	1.8	0.08	2.6	2.5	0.6
Benzo[ghj]perylene	<DL	1.1	na	<DL	<DL	na
Coronene	<DL	<DL	na	<DL	<DL	na
Sum PAH	67.0	126.9	**0.00**	19.4	17.3	0.1

The *n*-alkanes and the branched alkanes (Table 4) showed the typical pattern of CS as already described [32]. Alkanes with odd carbon numbers are mainly formed, in particular with C-numbers C27–C33. For branched alkanes, an alternation can be observed between odd-C-numbered iso-alkanes and even-C-numbered anteiso-alkanes. The most abundant iso-alkanes were isononacosane (i-C29), isohentriacontane (i-C31) and isotritriacontane (i-C33), whereas the most abundant anteiso-alkanes were anteisotriacontane (a-C30) and anteisodotriacontane (a-C32). In contrast with the PAHs, significantly higher amounts of alkanes in sidestream CS were only found at 500 mg/m^3^ TPM.

**Table 4:** Summary of *n*-alkanes and iso- and anteiso-alkanes in sidestream and mainstream CS *P* values were calculated using Student's unpaired *t* test. Values in bold are significant. na, not analysed.

	Sidestream			Mainstream		
Substance (μg/m^3^)	250 mg/m^3^	500 mg/m^3^	*P* value	250 mg/m^3^	500 mg/m^3^	*P* value
Nonadecane	90.3	97.9	0.52	193.5	60.1	na
Eicosane	82.7	163.5	**0.01**	43.1	59.3	0.14
Heneicosane	105.1	224.0	**0.01**	65.0	149.2	0.09
Docosane	111.7	269.5	0.10	75.8	64.1	0.29
Tricosane	86.8	237.6	**0.01**	73.1	57.5	0.15
Tetracosane	103.9	174.3	**0.03**	64.3	61.7	0.55
Pentacosane	189.4	379.9	**0.01**	147.8	117.3	**0.00**
Heptacosane	1006.2	2181.8	**0.00**	1040.6	825.1	**0.04**
Nonacosane	874.0	2170.7	**0.00**	1084.1	861.9	0.06
Triacontane	259.7	632.9	**0.00**	326.3	240.9	**0.02**
Hentriacontane	2966.9	7756.1	**0.00**	3677.9	2738.7	**0.03**
Dotriacontane	495.3	1199.4	**0.02**	695.3	513.9	**0.03**
Tritriacontane	1358.2	3609.9	**0.00**	2151.3	1515.5	**0.03**
Tetratriacontane	70.6	138.5	**0.01**	118.8	172.3	0.19
Pentatriacontane	41.5	78.7	0.06	57.2	48.5	0.13
Iso-nonacosane	169.4	452.3	**0.00**	222.7	192.4	0.34
Anteiso-triacontane	686.5	1749.8	**0.02**	838.4	837.7	1.00
Iso-hentriacontane	1069.2	3194.0	**0.00**	1578.0	1415.0	0.21
Anteiso-dotriacontane	1510.2	3639.7	**0.03**	2016.3	1918.0	0.71
Iso-tritriacontane	547.5	1440.8	**0.00**	735.6	650.8	0.22

### CC in the gas phase of CS

A total of 12 relevant CCs were identified in gas phase emissions from CS. Among those, acetaldehyde dominated in both sidestream and mainstream CS emissions with 250 and 500 mg/m^3^ TPM (Table 5). This predominance of acetaldehyde was already described in previous studies [33,34]. Unsaturated CCs such as acrolein, methacrolein and crotonaldehyde were also found in relevant concentrations in both types of CS.

**Table 5:** Carbonyl compounds in the gas phase of sidestream and mainstream CS *P* values were calculated using Student's unpaired *t* test.

	Sidestream			Mainstream		
Compound (μg/m^3^)	250 mg/m^3^	500 mg/m^3^	*P* value	250 mg/m^3^	500 mg/m^3^	*P* value
Formaldehyde	207.6	318.7	**0.02**	477.8	1027.5	**0.00**
Acetaldehyde	8000.0	11000.0	**0.00**	11900.0	32300.0	**0.00**
Propanal	330.5	492.2	**0.00**	415.4	1229.9	**0.00**
Acetone	2290.0	3120.0	**0.00**	2860.0	9700.0	**0.00**
Acrolein	139.7	263.6	**0.00**	336.1	746.6	**0.01**
Methacrolein	108.1	204.5	**0.00**	243.7	711.3	**0.00**
Crotonaldehyde	144.0	279.9	**0.00**	152.0	317.9	**0.00**
Isobutanal	128.3	228.2	**0.00**	131.2	318.2	**0.00**
Butanal	312.0	521.2	**0.01**	220.4	816.8	**0.00**
Butan-2-one	629.5	823.0	**0.02**	329.3	934.3	**0.00**
Isopentanal	241.1	432.2	**0.00**	221.5	548.9	**0.00**
Butan-2,3-dione	310.8	635.4	**0.00**	312.9	753.9	**0.00**

For most of the identified CCs the concentrations in sidestream CS were generally higher compared with mainstream CS and also showed greater than 2-fold increases with increasing TPM concentrations.

### BAL inflammation after sidestream CS exposure lacks neutrophilic influx

To determine inflammatory cell recruitment in BALs, differential cell counts on May–Grünwald–Giemsa-stained cytospins were performed.

**Figure 2:**
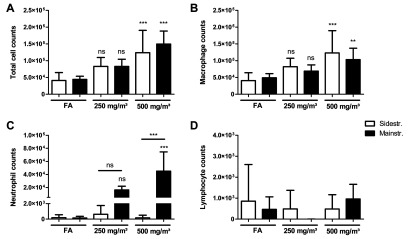
Characterization of BAL inflammation after acute smoke exposure reveals lack of neutrophilic influx in sidestream CS-exposed mice The lungs of mice exposed to CS for 3 days were lavaged with 4×0.5 ml aliquots of sterile PBS. Total cell counts were determined in a haemocytometer via Trypan Blue exclusion. Differential cell counts were performed using morphological criteria on May–Grünwald-Giemsa–stained cytospins (200 cells/sample). (**A**) Total cell counts. (**B**) Macrophages. (**C**) Neutrophils. (**D**) Lymphocytes. Results are means±S.D., one-way ANOVA following Bonferroni post-hoc test. **P<0.01 and ***P<0.001; FA, filter air; *n*=12. ns, not significant.

The 3 days of mainstream CS exposure significantly increased total cell numbers from control levels (44167±9601) to 83000±21389 for 250 mg/m^3^ TPM and 149750±38756 for 500 mg/m^3^ TPM respectively (Figure 2A). These mice showed BAL inflammation predominantly consisting of neutrophils and macrophages that were significantly elevated after exposure to 500 mg/m^3^ TPM (Figures 2B and 2C). Neutrophil numbers increased from 152±203 in filtered air controls to 16238±5878 at 250 mg/m^3^ TPM and to 45158±29315 at 500 mg/m^3^. Macrophages increased from 48967±12636 in filtered air controls to 69080±18357 for 250 mg/m^3^ TPM and to 103523±33576 for 500 mg/m^3^ TPM.

In contrast, BAL inflammation in the sidestream model was dominated by macrophages, leading to significantly elevated total cell and macrophage numbers after CS exposure to 500 mg/m^3^ TPM compared with control animals (Figures 2A and 2B). Total cell counts increased from 41071±23579 in controls to 83000±26609 in 250 mg/m^3^ TPM and to 123889±66628 in 500 mg/m^3^ TPM respectively. Macrophage numbers increased from 40657±23659 in controls to 81881±25488 in 250 mg/m^3^ TPM and to 123240±66195 in 500 mg/m^3^ TPM. BAL neutrophils remained unchanged after exposure to both sidestream CS concentrations (Figure 2C), leading to a significant difference in neutrophil cell counts between animals exposed to 500 mg/m^3^ mainstream CS compared with 500 mg/m^3^ sidestream CS.

As expected, lymphocyte numbers did not show any increase in both models compared with filtered air control animals (Figure 2D), confirming previous findings of two phases of inflammatory responses, with elevated lymphocyte levels starting after 1 month of CS exposure [12–14].

In sum, these results demonstrate that: (i) the CS concentration in the exposure chamber determines inflammatory cell recruitment to the alveolar lumen; and (ii) after sidestream CS exposure, a neutrophilic influx is not detectable.

### BAL inflammatory cytokines and chemokines are only increased after mainstream CS exposure

The secretion of a broad variety of inflammatory cytokines and chemokines into the airway lumen was analysed by sampling BAL fluid via a magnetic bead-based multiplex assay.

**Figure 3:**
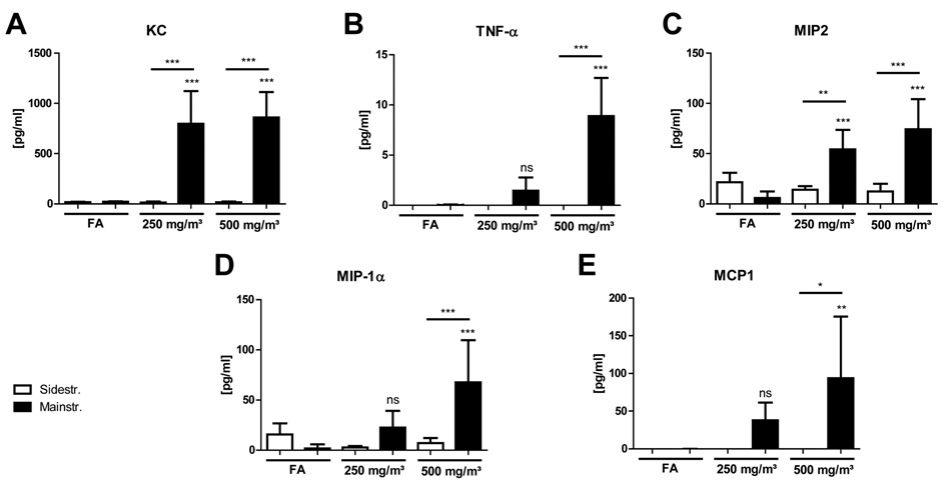
BAL inflammatory cytokine secretion after acute smoke exposure is only increased in mainstream CS-exposed mice Concentrations of secreted cytokines KC (**A**), TNF-α (**B**), MIP-2 (**C**), MIP-1α (**D**) and MCP1 (**E**) in BAL after 3 days of CS exposure were determined using a magnetic bead-based multiplex assay. For this assay, BAL fluid was concentrated (10×) by ultrafiltration in centrifugal filter devices. Results are means±S.D., one-way ANOVA following Bonferroni post-hoc test. *P<0.05, **P<0.01 and ***P<0.001; FA, filter air; *n*=12. ns, not significant.

Significantly up-regulated inflammatory mediators were only observed in BAL of mice exposed to mainstream CS compared with filtered air controls (Figure 3). Specifically, KC (CXCL1), TNF-α, MCP-1 (CCL2), MIP2 (CXCL2) and MIP-1α (CCL3), all of which are involved in innate immune cell activation and recruitment, were detected and found to be induced predominantly by the higher CS concentration of 500 mg/m^3^ TPM. In the sidestream model, no up-regulation was seen for any of the cytokines analysed in BAL as well as in the lung tissue homogenates (results not shown) of CS-exposed mice, explaining the significant differences in cell recruitment observed between animals exposed to mainstream CS compared with sidestream CS.

These results indicate a strong acute inflammatory response in the mainstream CS model, whereas there is no increased cytokine secretion after sidestream exposure for the markers analysed.

### Lung tissue inflammation is more prominent in mainstream CS-exposed mice

Lung tissue inflammation was further investigated by analysing mRNA expression levels of inflammatory target genes KC (CXCL1), TNF-α, MIP2 (CXCL2), MMP12 and CD68 in homogenized lung tissue.

**Figure 4:**
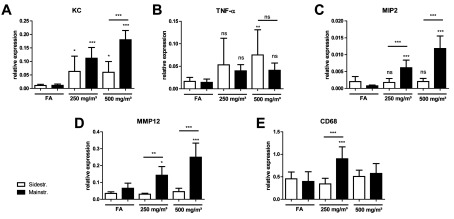
Gene expression profile in lung tissue after acute smoke exposure shows more prominent inflammation in mainstream CS-exposed mice mRNA expression levels of target genes KC (**A**), TNF-α (**B**), MIP-2 (**C**), MMP12 (**D**) and CD68 (**E**) in comparison with housekeeping control HPRT-1 were determined via quantitative real-time PCR using cDNA synthesized from lung tissue homogenate. Primers used are listed in Table 1. Relative mRNA expression of a gene is given as 2^−Δ*C*_t_^ (Δ*C*_t_=*C*_t_target__−*C*_t_reference__), relative changes compared with control are 2^−ΔΔ*C*_t_^ values (ΔΔ*C*_t_=Δ*C*_t_treated__−Δ*C*_t_control__). Results are means±S.D., one-way ANOVA following Bonferroni post-hoc test. *P<0.05, **P<0.01 and ***P<0.001; *n*=12. FA, filter air; *n*=12. ns, not significant.

In the mainstream CS model, mRNA levels for KC (CXCL1), MIP2 (CXCL2) and MMP12 were significantly increased after exposure of mice to CS concentrations of 250 and 500 mg/m^3^ TPM compared with control animals (Figures 4A, 4C and 4D). Interestingly, CD68 only showed elevated mRNA expression after CS exposure to 250 mg/m^3^ TPM (Figure 4E), whereas TNF-α did not increase at both concentrations (Figure 4B).

Mice exposed to sidestream CS also exhibited higher KC mRNA levels for both CS concentrations compared with control animals (Figure 4A). In contrast with the mainstream model, TNF-α showed elevated mRNA expression after CS exposure to 500 mg/m^3^ TPM (Figure 4B). However, MIP2 (CXCL2), MMP12 and CD68 did not increase at both concentrations (Figures 4C–4E). Significant differences in mRNA levels between animals exposed to mainstream CS compared with sidestream CS were observed for KC, MIP2 (CXCL2), MMP12 and CD68, mostly at higher CS concentrations of 500 mg/m^3^ TPM.

These results demonstrate that the strong acute inflammation observed in BAL (with increases in inflammatory cell recruitment and cytokine/chemokine secretion) after mainstream CS exposure was concomitant with increases in mRNA expression levels of inflammatory marker genes in lung tissue.

**Figure 5:**
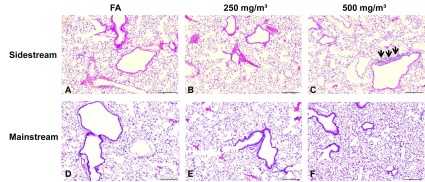
HE staining of lung tissue slides from mice exposed to filter air (A and D), sidestream (B and C) and mainstream (E and F) CS Exemplary micrographs revealed marginal inflammatory cell infiltrates after sidestream exposure to a CS concentration of 500 mg/m^3^ TPM (black arrows). Magnification 20×, scale bar 200 μm. FA, filter air.

Despite the findings of elevated mRNA expression of several proinflammatory genes, H&E staining of lung tissue from both models revealed only marginal inflammatory cell infiltrates after sidestream exposure to a CS concentration of 500 mg/m^3^ TPM (Figure 5). Furthermore, we did not observe any differences in lung function between control animals and CS-exposed mice from both exposure models (results not shown).

### Sidestream CS exposure leads to higher CO concentrations in the exposure chamber

CO, a gas derived from partial, i.e. incomplete, oxidation of carbon-containing compounds, is not only known to have toxic effects [35], but has revealed an important biological activity as a signalling molecule with protective actions against apoptosis and endothelial oxidative damage [36]. Therefore CO concentrations in the exposure chamber were constantly monitored during smoke exposure using a CO meter.

**Figure 6:**
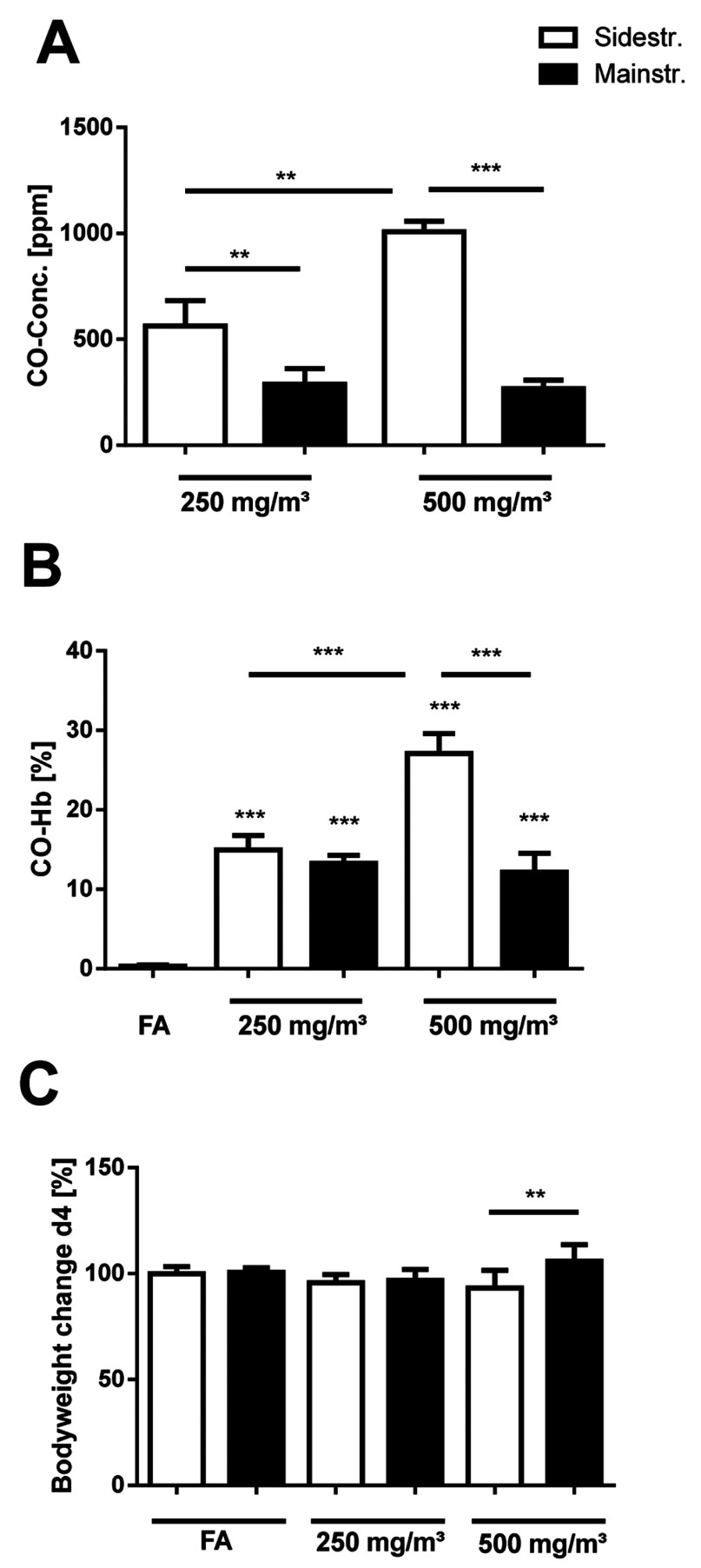
CO concentrations in the exposure chamber, levels of arterial blood CO–Hb and body weight changes in mice after acute smoke exposure (**A**) CO concentrations in the exposure chamber were monitored by using a GCO 100 CO Meter (*n*=4 measurements during one exposure cycle). (**B**) Levels of arterial blood CO-Hb in a subgroup of mice (*n*=4) 30 min after CS exposure were determined by retro-orbital blood sampling and analysed in an ABL80 FLEX blood gas analyser. (**C**) Body weight changes of CS-exposed mice on day of analysis (day 4); *n*=12. Results are means±S.D., one-way ANOVA following Bonferroni post-hoc test. **P<0.01 and ***P<0.001. FA, filter air; *n*=12. ns, not significant.

Mainstream CS of 250 and 500 mg/m^3^ TPM caused CO concentrations of 266±42 and 288±74 ppm, respectively (Figure 6A). On the other hand, sidestream CS exposure led to significantly higher CO levels in the exposure chamber compared with mainstream CS, with 563±119 ppm for 250 mg/m^3^ TPM and 1008±49 ppm for 500 mg/m^3^ TPM.

To determine the effects of CO levels on mice in the exposure chamber, arterial blood CO-Hb was measured in a subgroup of mice (*n*=4), 30 min after the last exposure. All mice tolerated CS-mediated CO concentrations without any sign of toxicity. In the mainstream model, arterial blood CO-Hb levels in mice exposed to 250 and 500 mg/m^3^ TPM increased from control values (0.3±0.2%) in filtered air animals to 13.2±1.1 and 12.2±2.4% respectively (Figure 6B). This is consistent with the similar levels of CO observed in the exposure chamber for both concentration levels of mainstream CS. Sidestream CS exposure to 250 mg/m^3^ TPM showed 15.0±1.8% at similar increases in CO-Hb compared with mainstream CS, in spite of almost 2-fold higher CO levels. In contrast, sidestream CS of 500 mg/m^3^ TPM was associated with significantly higher CO-Hb levels (27.1±2.5%) in mice compared with 500 mg/m^3^ mainstream CS (12.2±2.4%).

During the 3 days of smoke exposure, the body weight of all animals was monitored. Compared with controls, mice exposed to either main- or side-stream CS did not show any significant body weight changes during the whole exposure period (results not shown). However, a significant body weight difference (normalized to weight of each group on day 1) between animals exposed to 500 mg/m^3^ TPM of mainstream and sidestream CS was observed on the day of animal preparation (day 4), 24 h after the last CS exposure (Figure 6C), at 105.9±7.7% (mainstream) compared with 93.2±8.4% (sidestream).

These results demonstrate that sidestream CS exposure is associated with increased CO concentrations in the exposure chamber, thereby inducing elevated CO-Hb levels and body weight changes in mice especially for the CS concentration of 500 mg/m^3^.

### GM-CSF is only up-regulated in BAL and lung tissue of mainstream CS-exposed mice

Previous studies have described a role for GM-CSF in CS-induced pulmonary inflammation [37], and decreased GM-CSF production following CO exposure was observed in LPS (lipopolysaccharide)-stimulated macrophages [38]. Therefore mRNA and protein levels of GM-CSF in lung tissue and BALs were determined in the present study.

**Figure 7:**
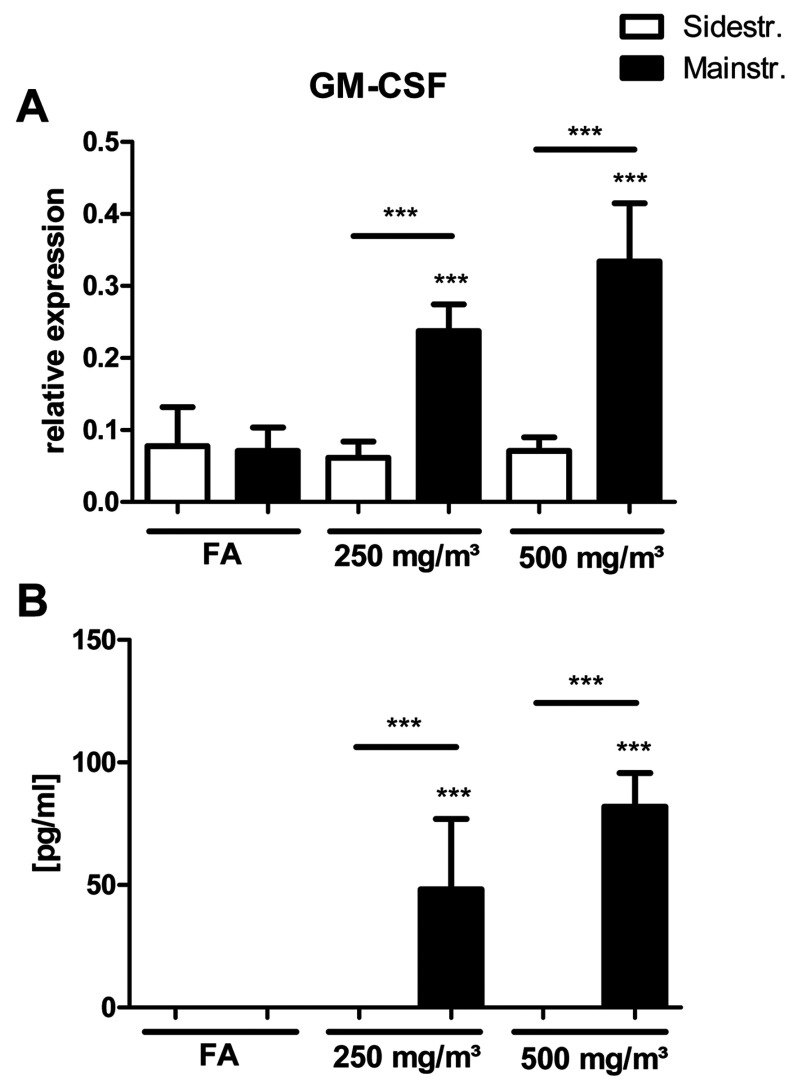
GM-CSF expression and secretion after acute smoke exposure is only up-regulated in lung tissue and BAL of mainstream CS-exposed mice (**A**) mRNA expression levels of GM-CSF in comparison with housekeeping control HPRT-1 were determined via quantitative real-time PCR using cDNA synthesized from lung tissue homogenate. Primers used are listed in Table 1. Relative mRNA expression is given as 2^−Δ*C*_t_^ (Δ*C*_t_=*C*_t_target__−*C*_t_reference__), relative changes compared with control are 2^−ΔΔC_t_^ values (ΔΔ*C*_t_=Δ*C*_t_treated__−Δ*C*_t_control__). (**B**) Concentrations of secreted GM-CSF in BAL after 3 days of CS exposure were determined using a magnetic bead-based multiplex assay. For this assay, BAL fluid was concentrated (10×) by ultrafiltration in centrifugal filter devices. Results are means±S.D., one-way ANOVA following Bonferroni post-hoc test. ***P<0.001. FA, filter air; *n*=12. ns, not significant.

mRNA expression levels of GM-CSF did not increase in sidestream CS-exposed mice, whereas the mainstream model shows a significant up-regulation for both CS concentrations (Figure 7A). The same was observed for secreted GM-CSF analysed in BAL of CS-exposed mice (Figure 7B), confirming the important role for GM-CSF in inflammatory cell recruitment in response to CS and its potential inhibition by CO.

## DISCUSSION

The present study aimed at investigating the composition of CS and the acute inflammatory response in the lungs of mice using two different experimental models of CS exposure for 3 days. The results presented here demonstrate that the composition of CS (TPM and CO concentration, chemical composition and size distribution of PM) determines the dynamics of inflammatory cell recruitment to the mouse lung.

CS is a toxic collection of more than 4800 chemicals and exceptionally rich in oxidants both in the gaseous and particle phases [39,40]. It is the major risk factor for cardiovascular diseases, atherosclerosis and lung diseases such as COPD and cancer. COPD is a disease characterized by airflow limitation caused by severe pathophysiological changes including chronic bronchitis, small airway remodelling, mucus production and the development of emphysaema [2]. In order to recapitulate these changes observed in patients suffering from COPD, CS-induced animal models are commonly used in laboratories around the world. Acute CS exposure has enabled researchers to study early and direct effects of smoking on inflammatory responses and cell recruitment, specifically innate immune cells and oxidative stress in the lung [20–22,24].

It is now generally accepted that the inflammatory response plays a major role in driving the pathophysiological changes observed in COPD [15,16]. Identical to observations in humans, CS constantly induces a neutrophil and macrophage inflammatory response in the lower respiratory tract of exposed animals (reviewed in [11,41]), and the acute reaction during the first week of CS exposure is dominated by a strong neutrophilic influx [12–14].

All studies looking at the dynamics of early lung immune cell reactions to CS used the mainstream model in acute settings of smoke exposure for a few days. In accordance with these studies, a strong acute inflammatory response characterized by neutrophilic influx, increased cytokine secretion (KC, TNF-α, MIP-2, MIP-1α and MCP1), proinflammatory gene expression (KC, MIP-2 and MMP12) and up-regulated GM-CSF production was observed in the mainstream CS model.

In contrast, only one study ever utilized the sidestream model in an acute setting, but did not focus on lung inflammation [24]. Howard et al. [24] investigated oxidative DNA damage via analysis of 8-OHdG (8-hydroxy-2′-deoxyguanosine) in mouse heart, liver and lung tissue and detected increased levels already after a single CS exposure for 30 min. In a chronic setting, Woodruff et al. [42] did not even observe neutrophil recruitment after exposures for 1, 2 and 3 months using the sidestream model, whereas macrophages were increased. Interestingly, the results of the present study show that there was a dampened inflammatory reaction after sidestream exposure, characterized by macrophages and slight increases in TNF-α mRNA expression.

In response to CS, neutrophils and macrophages release cytokines and proteolytic enzymes and generate oxidants, thereby causing tissue damage and perpetuating inflammation and immune reactions [41]. Gene expression profiling also revealed an early up-regulation of stress response and inflammation markers after acute CS exposure [13]. The proteolytic enzymes MMP12 and NE were even required for inducing experimental emphysaema after prolonged exposure [17,18]. CS-exposed MMP12-knockout mice failed to recruit macrophages and did not develop lung destruction [17], whereas NE was necessary for neutrophil and monocyte recruitment as well as for the activation of MMP12 after CS exposure [18].

Several studies characterized the inflammatory response after gradually increasing the exposure time and dose in the acute mainstream model [20–22]. Stevenson et al. [20] described a time- and dose-dependent increase in neutrophil chemokines, specifically CXCR2 ligands in the rat lung, which was mirrored by neutrophil infiltration. The work by Vlahos et al. [21] detected mRNA up-regulation of chemokines such as MIP-2 and MCP1, inflammatory mediators such as TNF-α, the leucocyte growth and survival factor GM-CSF and matrix degrading MMPs 9 and 12. Morris et al [22] confirmed time- and dose-dependent neutrophil increases in four different mouse strains, with highly up-regulated KC and MMP12 levels. The results of the present study also showed an mRNA up-regulation of CXCR2 ligands KC and MIP-2 as well as MMP12 after mainstream CS exposure. An increase in cytokine secretion was observed for KC, TNF-α, MIP-2, MIP-1α and MCP1.

The differences seen between sidestream and mainstream CS could be related to the biologically relevant, i.e. lung deposited PM dose. The observed smaller particle size in sidestream relative to mainstream CS was translated into a difference in the lung deposited dose using *in silico* models for particle deposition in the murine lung [31]. For a given TPM level, the lung-delivered PM dose of sidestream CS was approximately two times higher than for mainstream CS (see Table 2). Despite the enhanced biologically effective dose for sidestream CS conditions, the biological response for a given TPM level was less pronounced compared with the mainstream model. Thus, the pulmonary dose of PM cannot account for the inhibited inflammatory response in the sidestream CS model.

Besides the lung-delivered dose, the chemical composition of CS might influence the observed biological reactions. Mainstream and sidestream CS exhibit differences in the chemical profile, which is particularly related to the different combustion conditions during puffing and smoldering. Therefore the toxicological impact of mainstream and sidestream CS most probably differs as well. Sidestream CS is formed under more oxygen-deficient conditions at lower temperatures compared with mainstream CS. Large differences are obtained for the yields of toxic gaseous compounds such as acrolein, butadiene or benzene and CO, which are much higher for sidestream CS [43,44]. In addition, sidestream CS is characterized by significantly higher relative concentrations of nitrogen-containing compounds (e.g. ammonia or aniline- and pyridine-derivatives) [44], whereas mainstream CS exhibits higher concentrations of nitric oxide.

In the present study, CS-associated PAHs and alkanes were analysed. PAHs are a product of incomplete combustion as well as pyrolysis processes. Humans are mainly exposed to PAHs via inhalation of aerosols, such as CS particles. Animal testing has shown that many PAHs are potentially carcinogenic as PAH molecules bind to the AH (aromatic hydrocarbon) receptors. The enzymatic degradation yields in phenols and epoxides are caused by cytochrome P-450 dependent oxidases. The epoxy metabolites are partially transferred to dihydrodiol epoxides before excretion. Owing to ring-opening reactions, these epoxy derivatives can bind to DNA bases [45]. The knowledge of acute toxicity is sparse, but chronic health effects are to be expected, including lung, immune and reproduction toxicity. Furthermore, the total amount of alkanes far beyond 1 mg/m^3^ can cause irritations of the respiratory tract. The concentrations of *n*-alkanes and iso- and anteiso-alkanes in the 500 mg/m^3^ sidestream CS samples (0.75–8.53 ng/m^3^) were higher than those measured in 250 mg/m^3^ sidestream CS samples (0.77–1.51 ng/m^3^). Therefore differences in PM composition depending on the TPM level are expected. However, alkane as well as PAH concentrations only increased with higher TPM levels of sidestream CS, indicating the typically lower combustion temperatures and subsequently less complete combustion in sidestream CS conditions [44].

Sidestream CS of 250 mg/m^3^ and 500 mg/m^3^ TPM contained 3.4-fold and 7.2-fold higher PAH levels than mainstream CS respectively. The observed high concentrations of PAHs in sidestream CS indicate that high amounts of PAHs are formed during the smoldering phase of cigarette smoking when no puff is made [44]. In contrast with sidestream CS, the availability of oxygen and high temperatures during a puff of mainstream CS results in a more complete combustion of organic compounds yielding lower concentrations of PAHs. These results show the importance of sidestream CS for public health implications. However, the higher concentrations of PAHs and alkanes in sidestream CS were not reflected in enhanced biological responses in our model, suggesting a significant role of gas-phase components especially for sidestream CS.

CCs are important gaseous constituents of CS and some are toxic and carcinogenic or mutagenic to humans. They are highly reactive towards nucleophilic molecules in the cell such as DNA, proteins, aminophospholipids and glutathione and thereby contribute to the development of smoking related diseases [46,47]. The findings of the present study are in line with previous observations that higher concentrations for CCs such as acetaldehyde, acrolein, methacrolein and crotonaldehyde in the sidestream model might be caused by the incomplete combustion of sidestream CS as already described above.

Acrolein was described as effective in eliciting IL-8 release in pulmonary cells thereby contributing to neutrophil chemotaxis and activation [48,49]. Despite the higher production of acrolein in sidestream CS, we did not detect any appreciable level of neutrophils. Notably, this unresponsiveness needs further analysis. However, the dampened inflammatory response observed in the sidestream model might be related to elevated CO concentrations in the exposure chamber resulting in significantly higher levels of arterial blood CO-Hb in CS-exposed mice at 500 mg/m^3^ TPM compared with mainstream CS. Recent reviews pointed out the anti-inflammatory effects of CO in a multitude of *in vitro* studies and animal models of inflammation, indicating a therapeutic potential [36,50]. Besides its known toxic effects, CO has revealed an important biological function by protecting against apoptosis and endothelial oxidative damage. Recently, CO was shown to mediate anti-apoptotic effects of HO (haem oxygenase)-1, an isoform of the enzyme HO involved in the oxidative degradation of haem [51]. Moreover, CO as one of the three main byproducts of the catabolism of haem by HO altered iron homoeostasis in the lung thereby reducing oxidative stress and proliferation of bronchial epithelial cells [52].

An important study recently showed a protective effect of inhaled CO during pulmonary inflammation after LPS challenge [53]. CO administered to mice both before and after intratracheal instillation of LPS was effective in reducing neutrophil recruitment to the lung due to decreased bone marrow mobilization of leucocytes. The authors speculated that the effect on neutrophil mobilization occurred via decreased production of GM-CSF following CO exposure. This mechanism was already described in LPS-stimulated murine macrophages [38]. HO-1 overexpression as well as CO exposure inhibited LPS-induced GM-CSF production in macrophages via NF-κB (nuclear factor κB) inhibition, with NF-κB as the transcriptional regulator of GM-CSF.

As shown in Figure 7 GM-CSF levels for the sidestream CS model are significantly lower than for the mainstream model. GM-CSF might have an important role in CS-related lung diseases because it functions as a leucocyte growth, activation and survival factor [54]. It promotes proliferation and differentiation of haematopoietic progenitors into neutrophils and macrophages and is produced by many structural and inflammatory cells. Furthermore, GM-CSF acts as a direct neutrophil chemotactic factor and may increase neutrophil survival in the respiratory tract [55,56]. Vlahos et al. [37] demonstrated that neutralizing GM-CSF inhibited CS-induced lung inflammation by reducing BAL neutrophils and macrophages as well as TNF-α, MIP-2 and MMP12 mRNA expression. Possible mechanisms in this context were increased apoptosis of neutrophils and macrophages, because it was already shown that GM-CSF inhibits neutrophil apoptosis [57]. Furthermore, the effects of anti-GM-CSF treatment on MIP-2, another potent neutrophil chemotactic factor, could also account for the inhibited inflammation. The contribution of CXCR2-mediated signalling to CS-induced lung inflammation characterized by neutrophil influx was determined by treatment of mice with an inhibitor of CXCR2 [58]. A further study blocking GM-CSF receptor also described attenuated neutrophil influx in CS-exposed mice [59], confirming the major role of GM-CSF in CS-induced pulmonary inflammation.

Thus, the results of the present study are consistent with the hypothesis that in the sidestream CS model, high levels of CO and subsequently higher levels of arterial blood CO-Hb led to inhibited production and release of GM-CSF and CXCR2 ligands KC and MIP-2. However, despite the lack of difference in CO-Hb at 250 mg/m^3^ between the two models a largely different inflammatory response was observed. This might also point to effects of other gas phase components aside from CO that could significantly alter the inflammatory response in the lung. However, despite the findings of a dampened inflammatory response, it must be noted that inhaled fresh sidestream CS is approximately four times more toxic per gram TPM than mainstream CS [23]. In animals exposed to whole sidestream CS, sensory irritation and respiratory tract epithelium damage increases with longer exposures. However, the results of the present study show that the enhanced toxicity of TPM from sidestream CS was rather repressed by the gas-phase effects.

In summary, we demonstrated that significant differences exist between two mouse models of acute smoke exposure. Mainstream CS induced a strong acute inflammatory response and led to increased levels of neutrophils and related proinflammatory markers. In contrast, sidestream CS exposure only caused a dampened inflammatory reaction, with slightly elevated macrophage numbers. The different responses between the two models were most probably related to elevated CO concentrations, which inhibited early inflammatory responses in the sidestream CS model. The COPD mouse models for acute mainstream and sidestream CS exposure (i.e. also environmental CS) described in the present study are potentially interesting for evaluating the toxicological and health effects of other aerosol sources, such as emissions from house heating (e.g. wood stoves) or traffic-sources (e.g. trucks, cars or ships).

We conclude that a detailed analysis of physico-chemical and biological data generated from acute CS models can provide important information for potential therapeutic interventions at early stages of smoke exposure.

## CLINICAL PERSPECTIVES

• Initial inflammatory processes might contribute to COPD pathogenesis at an early stage of smoke exposure, but different animal CS exposure systems have been established and employed in acute and chronic COPD studies. In order to elucidate potential differences between mainstream and sidestream CS exposure, we investigated the CS composition and the acute inflammatory response in the lungs of mice after 3 days of CS exposure.
• We observed a strong acute inflammatory response characterized by neutrophilic influx, increased cytokine secretion and proinflammatory gene expression, and up-regulated GM-CSF production in the mainstream CS model, whereas there was a dampened inflammatory reaction after sidestream exposure, most probably caused by elevated CO concentrations.
• This comparison might be useful for the interpretation of data from the various CS mouse models and will potentially impact on therapeutic intervention studies starting at the early stages of smoke exposure.
